# Evolution of sexually-transferred steroids and mating-induced phenotypes in *Anopheles* mosquitoes

**DOI:** 10.1038/s41598-019-41094-4

**Published:** 2019-03-15

**Authors:** Emilie Pondeville, Nicolas Puchot, Michael Lang, Floriane Cherrier, Francis Schaffner, Chantal Dauphin-Villemant, Emmanuel Bischoff, Catherine Bourgouin

**Affiliations:** 1 CNRS, UMR2000, Institut Pasteur, 75724 Paris, Cedex 15 France; 2 MRC-University of Glasgow Centre for Virus Research, G61 1QH Glasgow, Scotland; 3 Functional Genetics of Infectious Diseases, Institut Pasteur, 75724 Paris, Cedex 15 France; 4 Institut Jacques Monod, CNRS, UMR7592, Université Paris Diderot, Sorbonne Paris Cité, Paris, France; 5 Francis Schaffner Consultancy, Riehen, Switzerland; 6 Swiss National Centre for Vector Entomology, Institute of Parasitology, Vetsuisse Faculty, University of Zürich, Zürich, Switzerland; 7 Université Pierre et Marie Curie-Paris 6, CNRS, 7 quai Saint Bernard, 75005 Paris, France; 8 Institut Pasteur, Unit of Genetics and Genomics of Insect Vectors, 75724 Paris, Cedex 15 France

## Abstract

Human malaria, which remains a major public health problem, is transmitted by a subset of *Anopheles* mosquitoes belonging to only three out of eight subgenera: *Anopheles*, *Cellia* and *Nyssorhynchus*. Unlike almost every other insect species, males of some *Anopheles* species produce steroid hormones which are transferred to females during copulation to influence their reproduction. Steroids are consequently a potential target for malaria vector control. Here, we analysed the evolution of sexually-transferred steroids and their effects on female reproductive traits across *Anopheles* by using a set of 16 mosquito species (five *Anopheles*, eight *Cellia*, and three *Nyssorhynchus*), including malaria vector and non-vector species. We show that male steroid production and transfer are specific to the *Cellia* and therefore represent a synapomorphy of this subgenus. Furthermore, we show that mating-induced effects in females are variable across species and differences are not correlated with sexually-transferred steroids or with *Anopheles* ability to transmit human malaria. Overall, our findings highlight that *Anopheles* mosquitoes have evolved different reproductive strategies, independently of being a malaria vector or not.

## Introduction

*Anopheles* mosquitoes are mostly known for their ability to transmit malaria parasites to humans with more than 200 million cases reported and an estimated 445000 deaths in 2016^1^. Among 475 species named in this genus, 41 have been classified as dominant vector species (DVS) of human malaria^2–5^. The *Anopheles* genus is further subdivided into eight subgenera of which three (*Anopheles*, *Cellia*, and *Nyssorhynchus*) contain all known DVS of human malaria^4^. The first essential condition to be a vector of human malaria is that the mosquito, after ingestion of infected blood, is permissive to the development of the parasite until the production of the infective sporozoites, a feature termed “vector competence”. The efficiency of malaria transmission by *Anopheles* mosquitoes in natural settings, named “vectorial capacity”, depends on vector competence but also on many other factors including the size of mosquito populations. Therefore mosquito reproduction and fecundity can affect malaria transmission by *Anopheles*.

Unlike vertebrates, it was considered that insect adult males do not produce significant amounts of steroid hormones until it was shown that males of *Anopheles gambiae*, the main vector of human malaria in Africa, produce and transfer high quantities of 20-hydroxyecdysone (20E) to females during copulation^6^. As ovarian steroids produced after blood feeding trigger egg development^7,8^, sexual transfer of steroids by males likely represents a nuptial gift that affects female reproduction in the malaria vector. Consistent with this, sexually-transferred steroids were shown to induce refractoriness to further copulation and to stimulate oviposition in *An. gambiae* females^9^. Interestingly, a further study examining 20E production by males in nine species and the effect of mating in three of them suggested that sexually-transferred steroids and associated mating-induced phenotypes (refractoriness to mating, increased egg development and oviposition) may have shaped the ability of DVS to transmit human malaria^10^. In the present work, we investigated the evolutionary history of male steroid production using a large set of mosquito species belonging to the three *Anopheles* subgenera (five *Anopheles*, eight *Cellia*, three *Nyssorhynchus*) that contain all known DVS of human malaria. We further analysed post-mating effects on female reproductive traits whether or not the cognate males produce steroids. Our results show that i) production of steroids in male mosquitoes is restricted to the *Cellia* subgenus, and ii) the effects of mating on female reproductive potential vary across species of the three *Anopheles* subgenera and this is not correlated with male steroid production. Overall our data highlight that *Anopheles* mosquitoes have evolved different reproductive strategies independently of sexually-transferred steroids and of their ability to transmit malaria.

## Results

### Male steroid production is specific to the *Cellia* subgenus

**Figure 1:**
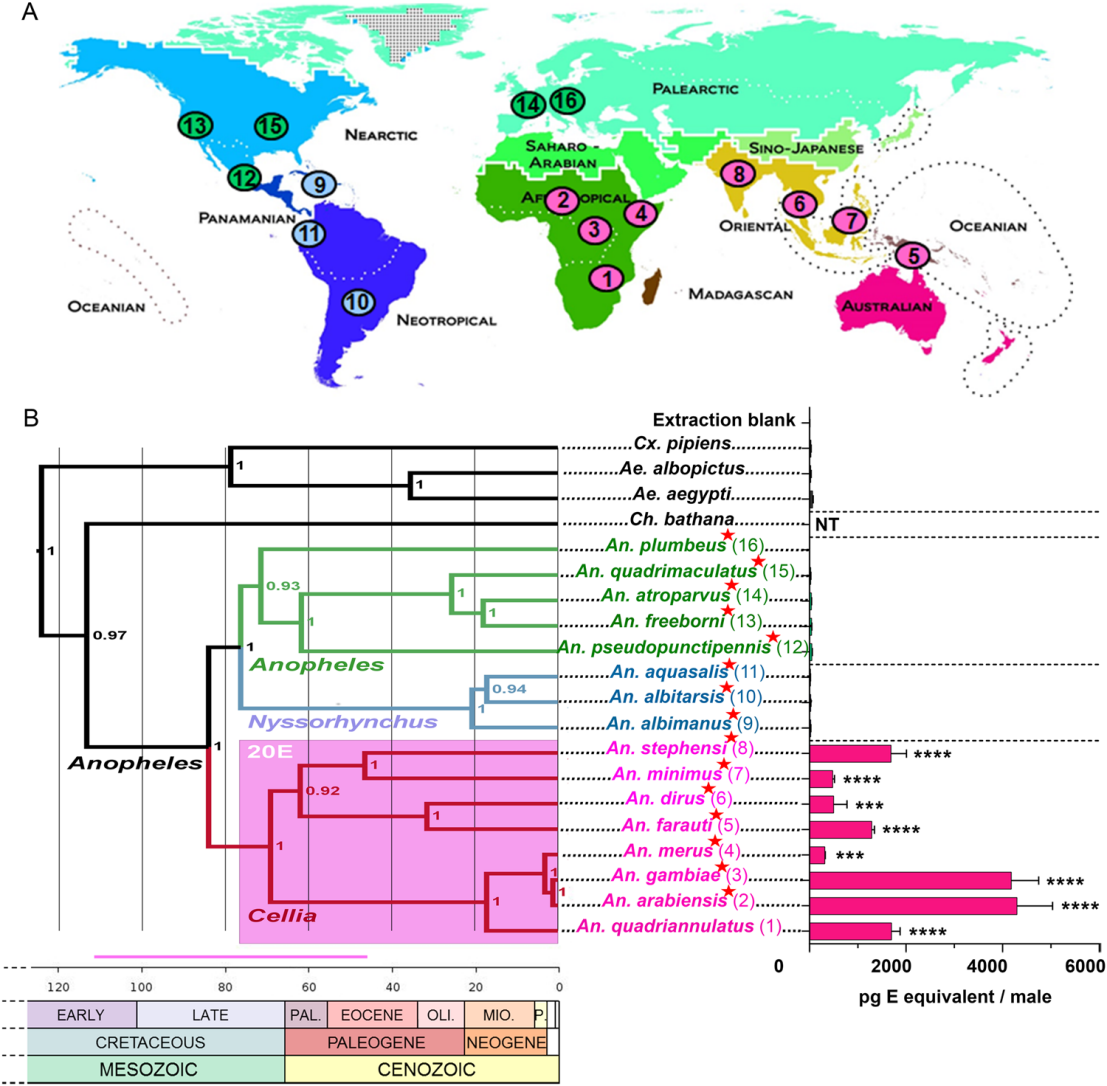
Distribution, phylogenetic relationships of mosquitoes and steroid production in mosquito adult males. (**A**) Present geographical distribution of the 16 mosquito species (*Anopheles* genus) analysed in this study represented on a zoogeographical map (modified from Journal Science/AAAS). Species matching numbers are shown on panel B. Pink: *Cellia* subgenus, blue: *Nyssorhynchus* subgenus, green: *Anopheles* subgenus. (**B**) Bayesian phylogeny of 20 *Culicidae* species, 19 species tested for ecdysteroid male production plus *Chagasia bathana* (subfamily *Anophelinae*, genus *Chagasia*) used as outgroup for phylogenetic analyses. Dominant human malaria vectors are indicated by a red star. Time is represented in millions of years (Ma). Approximated node ages are detailed in Supplementary Table 1. Bayesian node support values are presented on the right side of each node. Ecdysteroid titers in whole 5-day-old virgin males are indicated on the right side of the tree. Results are expressed as mean +/− standard error of the mean (SEM) in pg E equivalents per male. Results were subjected to statistical analysis using Kruskall-Wallis test for nonparametric data followed by Dunn’s post-hoc  test (control group: Extraction blank). The indicated p values are those obtained with Dunn’s test (***p value < 0.001; ****p value < 0.0001). NT: not tested. Predicted lineages with significant male 20E production are shaded pink on the tree. The pink horizontal bar represents the minimum/maximum 95% highest posterior density interval estimated time in Ma for origin of male 20E production. The geological time scale is adapted from the Geological Society of America (http://www.geosociety.org/science/timescale/). The white coloured cases represent the quaternary period. PAL., Paleocene; OLI., Oligocene; MIO., Miocene; P., Pliocene.

We measured steroid titers in sexually mature virgin males from 19 different mosquito species. These mosquito species were selected within two *Culicidae* subfamilies, *Anophelinae* and *Culicinae*. Within the *Anophelinae* subfamily, 16 species distributed all over the world were chosen to cover three different subgenera (*i.e. Cellia*, *Nyssorhynchus* and *Anopheles*) of the *Anopheles* genus (Fig. 1A). In the *Culicinae* subfamily, *Aedes aegypti, Aedes albopictus* and *Culex pipiens* that are vectors of arboviruses were also analysed. As shown on Fig. 1B (right panel), male 20E production occurs only within the *Cellia* subgenus (*Anophelinae*) and is absent in the *Culicinae*. Interestingly, *Anopheles quadriannulatus* whose males produce similar levels of steroids compared to *Anopheles stephensi* is not a malaria vector unlike *An. stephensi* and other *Cellia* species investigated in the present study. Indeed, its refractoriness or low susceptibility to the human malaria parasite has been experimentally determined^11,12^, confirming epidemiological data^13^. Conversely, production of 20E was not detected in male mosquitoes from the two other *Anopheles* subgenera, *Anopheles* and *Nyssorhynchus* of which all tested members are registered as DVS and/or experimentally shown to be highly susceptible to *Plasmodium falciparum*^14,15^.

To uncover the evolutionary history and divergence time of male steroid production in mosquitoes, we analysed the phylogenetic relationships of the selected species. To this end, we used DNA sequences of partial regions of the coding sequence of mitochondrial genes (*COI*, *COII, ND5 and CYTB*) and nuclear genes (*g6pd* and *white* as well as ribosomal subunits *18S* and *28S*) from the 19 mosquito species plus *Chagasia bathana* (*Anophelinae* subfamily, *Chagasia* genera) as outgroup for *Anopheles* mosquitoes^16^. Phylogenetic analysis of the data set was performed by Bayesian inference (Fig. 1B left panel) and by maximum likelihood (Supplementary Fig. 1). Phylogenetic relationships inferred from these analyses resulted in different topologies mainly at the subgenus level and with varying node support values. In both analyses, the members of each subgenus formed monophyletic groups and the branching orders within the subgenus *Cellia* was identical. Our results are in agreement with those obtained by Sallum *et al*.^17^ with the *Cellia* clade being the outgroup of the subgenera *Anopheles* and *Nyssorhynchus* in the Bayesian approach while in the maximum likelihood approach, *Nyssorhynchus* is the outgroup of *Anopheles* and *Cellia*, also in agreement with recently published phylogenies^2,18,19^. The *Anopheles* subgenus has also been found by others to be a basal lineage to *Cellia* and *Nyssorhynchus*^20^. Thus, relationships between the different subgenera *Cellia*, *Nyssorhynchus* and *Anopheles*, are still not entirely resolved. The difficulty in determining the relationships among these subgenera might be due to limits and bias in gene and taxon sampling, but also to the different methods used^4,16^. This could also be due to the fact that the radiation of *Anopheles* and *Cellia* species happened roughly at the same time^18,20^.

From the Bayesian phylogenetic analysis and using fossil data, we obtained species divergence time estimates, which largely conform to recent phylogenies^18,20,21^ (Supplementary Table 1). The age of the last common ancestor of *Anopheles* genus is about 84.1 Ma (112.7–55.8, 95% highest posterior density [HPD] interval) and the most ancestral node within the *Cellia* subgenus is dated to 69.2 Ma (93.0–45.6, 95% HPD interval) (Fig. 1B, Supplementary Table 1). As males from all *Cellia* species tested so far have the ability to produce 20E while males from all species belonging to the other subgenera do not, according to the “law of parsimony” steroid production by mosquito males probably originated once in the ancestral *Cellia* lineage, at about 84.1–69.2 Ma (112.7–45.6, 95% HPD interval), *i.e*. during the late Cretaceous. Thus, steroid production by mosquito males is most probably a shared derived character from the last common ancestor of *Cellia* mosquitoes and represents as such a synapomorphy of this subgenus. As 20E production in males is private to the *Cellia* subgenus, which in addition forms a monophyletic group in the two phylogenetic approaches, the conflicting distribution of the outgroup lineages (*Anopheles* and *Nyssorhynchus*) does not alter the conclusion that steroid production by males most probably originated after the split between *Cellia* and these two other subgenera.

**Figure 2:**
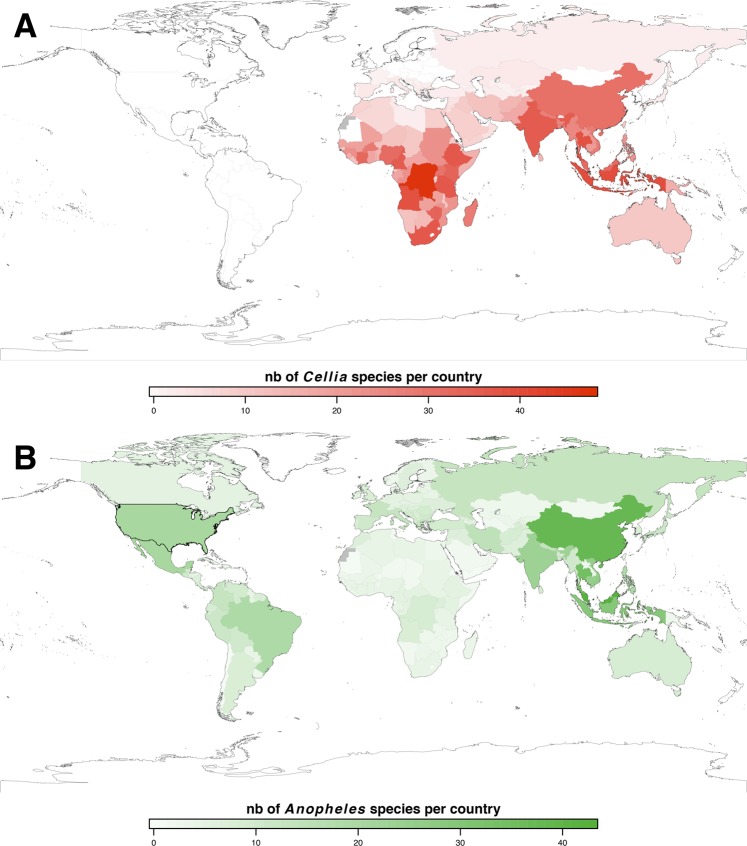
Present geographic distribution of species belonging to *Cellia* and *Anopheles* subgenera (*Anopheles* genus). Total numbers of mosquito species belonging to the *Cellia* (**A**, red) and *Anopheles* (**B**, green) subgenera per country (sourced from the Walter Reed Biosystematics Unit, http://www.wrbu.org/) are represented on world maps created with R. Numbers (nb) of mosquito species per country are represented by a coloured gradient as depicted under each map. Grey colour means no data are available for the country.

*An. stephensi* males transfer steroid to females upon mating (Supplementary Fig. 2) as do *An. gambiae* males^6^ and males of other *Cellia* species such as *Anopheles arabiensis* and *Anopheles dirus*^10^. This strongly suggests that transfer of steroids to females during mating is part of the “male 20E production” synapomorphy of *Cellia* mosquitoes. Our geographical mapping of nearly all contemporary mosquito species belonging to the *Cellia* subgenus (224 species) against the ones of *Anopheles* subgenus (184 species) (Fig. 2) suggests that the common ancestor of the *Cellia* subgenus diverged after separation of South America and Africa in agreement with previous observations^4,22^. This biogeographic calibration is consistent with divergence times obtained from our phylogenetic analysis placing the origin of steroid production by males of the *Cellia* subgenus around 84.1–69.2 Ma (112.7–45.6, 95% HPD interval), *i.e*. after the separation of South America and Africa, which started at least 100 Ma ago with no land bridge for about 80 to 50 Ma^23,24^.

### Mating-induced effects do not correlate with sexually-transferred steroids

**Figure 3:**
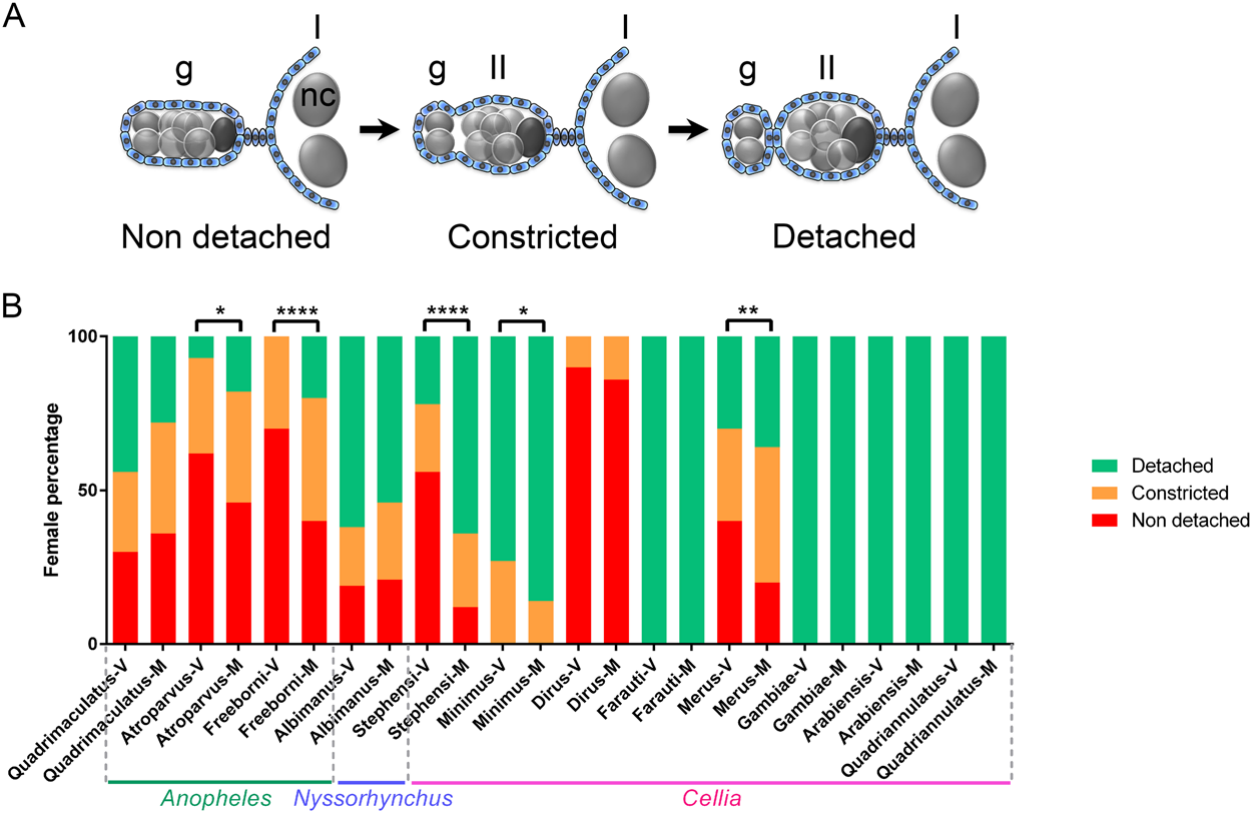
Secondary follicle detachment from the germarium in virgin and mated non blood-fed females from 12 *Anopheles species*. (**A**) Cartoon representing the detachment of the secondary follicle from the germarium. Follicular cells of somatic origin are coloured in blue. Germ cells (germline stem cells, developing cysts and nurse cells) are coloured in grey and the future oocyte of the secondary follicle in dark grey. g: germarium, I: primary follicle, II: secondary follicle, nc: nurse cells. (**B**) Secondary follicle detachment from the germarium in ovarioles of virgin (V) and mated (M) females. Secondary follicles are either detached from the germarium (detached, green), in the process of detachment (constricted, orange) or not yet detached (non-detached, red). Representative pictures of these three states are shown in Supplementary Fig. 3. The secondary follicles are significantly more detached in mated females compared to virgin females for *An. atroparvus* (p = 0.0226), *An. freeborni* (p < 0.0001), *An. stephensi* (p < 0.0001), *An. minimus* (p = 0.0228) and *An. merus* (p = 0.0072).

In female mosquitoes, each ovary is composed of 50 to 500 units called ovarioles that develop synchronously. At the time of adult emergence, each ovariole consists of a germarium and one follicle, called the primary follicle (see Fig. 3A). In some species, a secondary follicle is also present. Formation and detachment of follicles at this stage is thought to be controlled by steroids produced during the pupal stage. During two to three days after emergence and under the action of Juvenile Hormone (JH), primary follicles mature to a resting-stage in which they remain until females take a first blood meal. After blood meal ingestion, ovarian steroids trigger vitellogenesis of the primary follicles and egg development as well as the detachment from the germaria of new follicles, either secondary follicles if not yet present at emergence or tertiary follicles if secondary ones were already detached. Secondary follicles then grow to the previtellogenic resting stage, and develop after a second blood meal^25^.

To get a deeper understanding of the effect(s) of sexually-transferred steroids in *Cellia* female mosquitoes, we investigated the influence of mating on two events happening during the ovarian cycles which are known to be regulated by 20E. Firstly, injections of steroid hormones trigger follicle formation and detachment from the germarium in *An. stephensi* and *Ae. aegypti*^26,27^. Secondly, ovarian steroids produced upon blood feeding stimulate vitellogenesis and egg development^7,8,25,27,28^. We therefore compared follicle detachment (in females at the previtellogenic resting stage) and egg development (in blood fed females) between virgin and mated females from 12 *Anopheles* species (8 *Cellia*, 3 *Anopheles* and 1 *Nyssorhynchus*). As expected, mating induces the separation of the secondary ovarian follicle from the germarium in *An. stephensi* (Fig. 3, see also confocal pictures Supplementary Fig. 3), a *Cellia* species whose males produce and transfer steroids during mating. Mating also induces the separation of the secondary follicle in *Anopheles minimus* and *Anopheles merus*, although to a lesser extent. However, no secondary follicle detachment was observed after mating in *An. dirus*, a species for which none of the virgin females has the secondary follicle already detached. While multiple injections of steroids trigger the formation and detachment of the secondary and tertiary follicles within the same ovariole, in non-blood fed *Ae. aegypti*^26^, mating does not trigger detachment of tertiary follicles in *Cellia* species whose secondary follicles are already fully detached in virgin females (*Anopheles farauti*, *An. gambiae*, *An. arabiensis* and *An. quadriannulatus*). Mating also induces secondary follicle detachment in some species whose males do not produce steroids (*Anopheles atroparvus* and *Anopheles freeborni*), but not in others, even in species showing partial detachment of the secondary follicle in virgin females (*Anopheles quadrimaculatus* and *Anopheles albimanus*).

**Figure 4:**
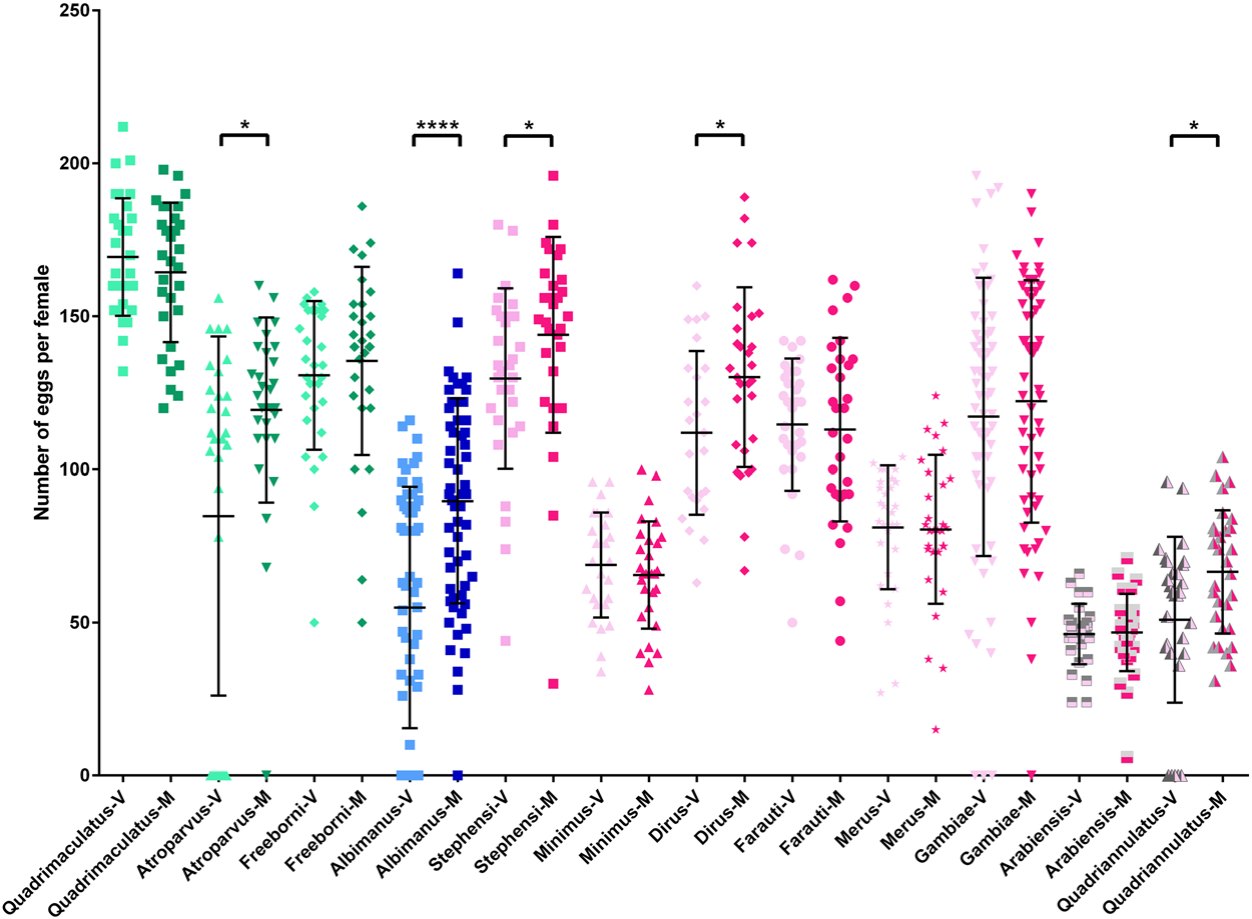
Egg development in virgin and mated blood-fed females from 12 species of *Anopheles* mosquitoes. Total number of eggs in virgin (V, light colours) and mated (M, dark colours) females 48 hours after blood feeding. Green: *Anopheles* subgenus, blue: *Nyssorhynchus* subgenus, pink: *Cellia* subgenus. Females from *An. atroparvus* (Mann-Whitney U = 295.5, p = 0.0214), *An. albimanus* (Mann-Whitney U = 941.5, p < 0.0001), *An. stephensi* (Mann-Whitney U = 232.5, p = 0.0235), *An. dirus* (Mann-Whitney U = 298, p = 0.0240) and *An. quadriannulatus* (Mann-Whitney U = 309.5, p = 0.0373) develop significantly more eggs when they are mated.

Similarly, mating increases the number of developing eggs in some mosquito species but not in others (Fig. 4). There is no correlation with the cognate male producing steroids, nor with the ability of virgin females to produce a low or a high number of eggs upon blood feeding (Fig. 4 and Table 1). As an example, mating increases egg development in *An. albimanus* (*Nyssorhynchus*), whose males do not produce steroids, but not in *An. gambiae* nor in *An. arabiensis* (*Cellia*). These results are in discrepancy with previous studies reporting that mating increases egg development in *An. gambiae* and *An. arabiensis*^10,29^ but not in *An. albimanus*^10,30^. These differences across studies are not due to the origin of the blood ingested by the females (animal or human) as similar results were obtained with *An. albimanus* and *An. gambiae* fed on mouse or human blood (Supplementary Fig. 4). Importantly, in *Cellia* species, the occurrence or absence of mating-induced phenotypes in females are not linked to the different quantities of steroids produced by the cognate males (Fig. 1 and Table 1) and likely transferred to females during mating as determined for *An. gambiae*, *An. stephensi*, *An. arabiensis* and *An. dirus* (Supplementary Fig. 2)^6,10^. Indeed, as depicted in Table 1, mating triggers both secondary follicle detachment and a rise in the number of developed eggs in *An. stephensi*, or only an increase in egg development in *An. dirus*, while this does not hold for *An. gambiae* and *An. arabiensis*, two species whose males produce and transfer higher amounts of steroids than *An. stephensi* and *An. dirus*.

**Table 1:** Summary of mating effect on follicle detachment and egg development in regard to malaria vector status and male steroid production in *Anopheles* species.

Subgenus	Species	DVS	Male steroid production	Follicle detachment	Egg development
*Anopheles*	*An. quadrimaculatus*	+	−	−	−
*Anopheles*	*An. atroparvus*	+	−	+	+
*Anopheles*	*An. freeborni*	+	−	+	−
*Nyssorhynchus*	*An. albimanus*	+	−	−	+
*Cellia*	*An. stephensi*	+	++	+	+
*Cellia*	*An. minimus*	+	+	+	−
*Cellia*	*An. dirus*	+	+	−	+
*Cellia*	*An. farauti*	+	++	−	−
*Cellia*	*An. merus*	+	+	+	−
*Cellia*	*An. gambiae*	+	+++	−	−
*Cellia*	*An. arabiensis*	+	+++	−	−
*Cellia*	*An. quadriannulatus*	−	++	−	+

Dominant vector species (DVS) of human malaria are signalled by a +. For male steroid production, relatively low titers (mean range 500 pg E equivalent per male) are indicated by +, medium (range [1000–2000] pg E equivalent per male) titers by ++, and high titers (above 3000 pg E equivalent per male) by+++. For follicle detachment and increase of egg development, − indicates no effect of mating and + indicates an effect of mating on either reproductive traits in females.

## Discussion

Since the discovery that *Anopheles gambiae* males were “unique” among insects by producing and transferring 20E to females^6^, steroids and steroid-signalling pathways became potential targets for malaria vector control^31,32^. Indeed, it was suggested that some post-mating responses such as refractoriness to further mating and increase in egg development only exist in *Anopheles* species whose males produce and transfer steroids to females and that may be linked to the ability of these mosquitoes to transmit human malaria^10^. However, a deep understanding of the selective forces driving reproductive strategy diversity and their functional consequences are critical for designing strategies for management of insect pests. Here, by analysing the evolution of steroid production and transfer by male mosquitoes, we reveal that this physiological trait is specific to the *Cellia* subgenus. The *Anopheles* species previously identified as transferring steroids indeed belong to the *Cellia* subgenus only. However, the analysis of a limited number of species could not reveal this synapomorphy^10^. Furthermore, by using a larger set of mosquito species we demonstrate that there is no strict correlation between the evolution of sexually-transferred 20E and the evolution of malaria transmission to humans (Table 1). Consistent with this, while we show that male steroid production and subsequent transfer to females is likely to have evolved only once in the common ancestor of the *Cellia* species, phylogenetic analyses on malaria mosquitoes support a convergent evolution with independent and repeated acquisitions of vectorial capacities in *Anopheles* mosquitoes even though losses also occurred^33–35^. Nevertheless, as sexually-transferred steroids influence egg production in some species, we cannot exclude they could impact malaria transmission by these species. This is also true for species not transferring steroids and for which mating influences egg production even though it is not yet known which sexually-transferred molecule mediates these effects.

While post-mating responses are rather conserved among insects, they are triggered by species-specific genes and signalling pathways due to the rapid evolution of insect reproductive systems^36^. For instance, *Ae. aegypti* males transfer JH to females upon copulation to trigger physiological and behavioural changes in mated females, whereas *Drosophila* flies transfer male accessory gland peptides such as Sex Peptide^37,38^. However, few data were available for *Anopheles* mosquitoes and it has been suggested that mating-induced changes, mediated by male steroids, do not exist in species whose males do not produce and transfer steroids. Importantly, our analysis demonstrates that identical post-mating responses increasing fecundity occur in *Cellia* species, but also in mosquito species whose males do not produce and transfer steroids such as in species of the *Anopheles* and *Nyssorhynchus* subgenera (Table 1). Injection of 20E into virgin females induces refractoriness to further mating in *An. gambiae* and *An. arabiensis* but not in *An. albimanus* which do not produce male 20E, linking male 20E transfer with monogamy (insemination by a single male) in the former species^9,10^. Similarly to the post-mating responses analysed in this study, monogamy is common in *Anopheles* mosquitoes, whether males produce steroids or not^39–42^. It is therefore likely that mosquitoes belonging to the *Anopheles* and *Nyssorhynchus* subgenera transfer other and not yet identified molecule(s) to directly or indirectly increase egg development, stimulate follicle detachment and induce refractoriness to further mating in mated females as sexually-transferred steroids do in *Cellia* species. For instance, it could be possible that in these species not transferring steroids, mating triggers the females to produce their own steroid hormones, which in turn would have the same effects as the sexually-transferred ones in *Cellia* species. As JH is transferred to females during mating in *Ae. aegypti* mosquitoes but also in the Lepidoptera *Heliothis virescens*^43^, sexual transfer of steroid hormones in other mosquito subgenera, genera or even insect orders not yet tested cannot even be excluded.

Overall, our data demonstrate that mating-induced phenotypes are variable among *Anopheles* species. These differences are independent of male ability to produce and transfer steroids to females and are not correlated with malaria vectorial capacity. In agreement with this, a recent study found that molecular pathways triggered by male 20E are different in *An. gambiae* and *An. coluzzii* females, members of the *Anopheles gambiae* complex that recently diverged and live in sympatry in many places in Africa^44^. While previous reports demonstrated that mating increases egg development in *An. gambiae* and *An. arabiensis* but not in *An. albimanus*^10,29,30^, our results show the opposite, *i.e*. mating increases egg development in *An. albimanus* but not in *An. gambiae* and *An. arabiensis*. These different results obtained for the same species across laboratories show that mating-induced phenotypes are possibly variable even among strains as already described for some *Aedes* species^45,46^. In the same way *An. coluzzii* males produce higher amounts of 20E under wet versus dry conditions^47^. It cannot therefore be excluded that male steroids transferred upon mating benefit reproduction of some *Cellia* species but only under certain environmental conditions. Different ecological pressures such as nutrition resources might also favour the maintenance and importance of sexually-transferred steroids, as shown for nuptial gifts in *Ae. aegypti*^48–50^. The reproductive capacity of mosquito females is affected by their nutrition at both the larval and adult stages. In *An. gambiae* (*Cellia*), *An. stephensi* (*Cellia*), *An. albimanus* (*Nyssorhynchus*) and *An. quadrimaculatus* (*Anopheles*), reserves acquired during larval development are lower and females invest less of their blood meal resources in oogenesis than in *Ae. aegypti*. Moreover, starvation during larval stages decreases female body size, which in turn decreases the volume of ingested blood and then the number of matured eggs^51^. As a consequence, wild female *Anopheles* are likely to seek multiple blood meals during a single gonotrophic cycle, especially the first one^52^. It is therefore possible that steroids transferred during mating in *Cellia*, as well as unidentified molecule(s) in *Nyssorhynchus* and *Anopheles* subgenera, constitute an adaptation within the *Anopheles* genera to enhance fertility and reduce the multiple seeking behaviour in case of poor larval reserves.

Apart from follicle detachment, increase in egg development and induction of refractoriness to mating, numerous other functions of steroids have been described in adult insects^53–56^. Thus, sexually-transferred steroids could mediate different functions with more or less direct benefit for the reproduction of *Cellia* females, due to the rapid evolution of reproductive systems between species. Sexually-transferred steroids could be preferentially used to control one or another female reproductive trait, and differently according to various reproductive strategies among species. If this is the case, this could explain why we could not see any effect of mating on follicle detachment and egg development in some species such as *An. gambiae* and *An. farauti*, or only an effect on egg development but not on follicle detachment such as in *An. dirus*. For this latter species, it cannot be excluded as well that another signal, such as the blood meal, is also required to trigger secondary follicle detachment. It is unlikely that the occurrence or absence of mating-induced effects are due to the quantity of steroids being transferred as for instance, males of *An. gambiae* produce and transfer to females much more steroids compared to *An. dirus*.

It remains open as what were the evolutionary forces that have initially promoted the acquisition and radiation of this presumably costly male steroid production and sexual gift to females in *Cellia* mosquitoes around 84.1–69.2 Ma. At this time, two main paleogeological events that had impacts on environmental conditions may have led to pressures driving the evolution of steroid production and transfer by males in *Cellia* species: (i) the Gondwana break up at 100 Ma with separation of South America and Africa^57^; (ii) the Cretaceous-Paleogene extinction event at 65.5 Ma^58,59^. Because such geographical isolation and environmental stresses are believed to drive traits contributing to animal species survival, it is likely that transfer of steroids by males to females have favoured *Cellia* species populations at this critical time.

## Material and Methods

### Mosquito species and rearing

Ecdysteroid production by sexually mature males was analysed in 19 mosquito species. Sixteen (16) species belong to the subfamily *Anophelinae* (*An. arabiensis*, *An. dirus*, *An. farauti*, *An. gambiae* form M, *An. merus*, *An. minimus*, *An. quadriannulatus, An. stephensi*, *An. albimanus*, *An. albitarsis*, *An. aquasalis*, *An. atroparvus*, *An. freeborni*, *An. plumbeus, An. pseudopunctipennis* and *An. quadrimaculatus*) and three species belong to the subfamily *Culicinae* (*Ae. aegypti, Ae. albopictus* and *Cx. pipiens*). Information on species is given in Supplementary Information. Mosquito larvae were reared at 27 °C in deionized water supplemented with minerals and fed on TetraMin Baby-E fish food from the day of hatching to the fourth larval instar supplemented with pieces of cat food. Male and female adults were maintained at 27 °C, under 68% relative humidity and a 12/12 h light/dark cycle, and provided free access to a 10% wt/vol sucrose solution for the first five days post-emergence (PE). Female mosquitoes (first gonotrophic cycle) were allowed to feed for 30 min on the blood of an anesthetized mouse or rabbit depending on mosquito species preference (see Supplementary Table 2). *An. plumbeus* L4 larvae and pupae were harvested from a natural pond in Switzerland and reared up to the adult stage at the Institut Pasteur. Mature males of *An. albitarsis* and *An. aquasalis* were kindly prepared and provided by D. Valle and L. Moreira (Fiocruz, Brazil) and *An. pseudopunctipennis* by F. Lardeux (IRD, Bolivia).

### Mosquito sampling, ecdysteroid extraction and quantification

To measure ecdysteroid titers in virgin adult males and females, males and females were separated on the day of adult emergence, transferred individually 5 days later in methanol and stored at −20 °C until ecdysteroid extraction. For the transfer experiments, *An. stephensi* males and females were separated on the day of adult emergence for 5 days to allow male sexual maturation. Mating experiments and sampling were performed as described in^6^. Total ecdysteroids from individual whole mosquitoes were extracted with methanol and re-dissolved in enzyme immuno assay (EIA) buffer. Empty tubes were treated similarly in parallel to be used as a negative control (referred as extraction blank). Ecdysteroids were quantified by EIA, with 20-hydroxyecdysone-2-succinate coupled to peroxydase as a tracer (dilution 1:100,000) and the L2 antiserum (gift from M. De Reggi (Marseille, France); dilution 1:100,000). Calibration curves were generated with ecdysone (E; 3,6–500 pg/tube) diluted in EIA buffer, and titers were expressed as E equivalents. Under these conditions, detection limit is 2 pg E equivalents. All measurements were performed in duplicate and the results are expressed as mean values ± SEM of several (n = 20 at least) independent samples and have been repeated on two independent cohorts of mosquitoes. Samples at or above the highest value of the calibration curve were diluted and quantified again. The intra- and inter-assay variation coefficients were 3,9% and 5,6%, respectively. For steroid titers in whole males from different species, data were subjected to statistical analysis using Kruskall-Wallis test for nonparametric data followed by Dunn’s post-hoc test (control group: extraction blank). The indicated p values are those obtained with Dunn’s test. For the transfer experiment in *An. stephensi*, results were subjected to statistical analysis using the Mann-Whitney test.

### Taxon sampling and DNA sequencing for phylogenetic analysis

We chose 20 *Culicidae* species for phylogenetic and comparative analysis. We selected 16 species of the genus *Anopheles (Anophelinae* subfamily) of which *An. arabiensis*, *An. dirus*, *An. farauti*, *An. gambiae*, *An. merus*, *An. minimus*, *An. stephensi*, *An. quadriannulatus*, *An. atroparvus*, *An. freeborni*, *An. plumbeus*, *An. pseudopunctipennis*, *An. quadrimaculatus*, *An. albimanus*, *An. albitarsis*, and *An. aquasalis*. As outgroups, we chose *Chagasia bathana* (*Anophelinae* subfamily, *Chagasia* genus), and 3 mosquito species belonging to the subfamily *Culicinae* with *Ae. aegypti*, *Ae. albopictus* (*Aedes* genus) and *Cx. pipiens* (*Culex* genus). Sequence data were generated for *An. aquasalis*, *An. atroparvus*, *An. merus* and *An. plumbeus*. Genomic DNA was obtained from single individuals using the DNeasy Blood and tissue kit (QIAGEN). A common set of molecular markers were chosen based on the availability of sequence data for *Ch. bathana*. Partial genomic regions of four nuclear genes (*g6pd, white, 18S and 28S*) and four mitochondrial genes (*COI*, *COII*, *ND5* and *CYTB*) were amplified by PCR with gene-specific or degenerate primers (sequences in Supplementary Table 3). For PCR amplifications, we used 0.4 µM oligonucleotides, 1 U GoTaq® DNA Polymerase (Promega) per 35 µl reaction volume, 2 mM MgCl_2_, and 200 µM dNTP and reactions were carried out using standard thermocycle conditions. PCR products were purified and Sanger-sequenced with gene-specific primers or with T7, SP6 universal primers at Cogenics (www.cogenics.com, Beckman Coulter, GenBank Accession Numbers in Supplementary Table 4). Sequence data of the remaining species were obtained from GenBank and VectorBase (Supplementary Table 4). Sequences were examined and aligned with Geneious 6.1.3 (Biomatters). Our dataset was not complete, we did not find or generate sequence data for 10 / 160 (8 genes X 20 species) gene specific sequences and among the rest 15 / 150 of sequences were only partially covering the locus. The missing sequence data represent 5.6% of the total dataset and was annotated as a “?” (missing value) in the alignments for phylogenetic analysis. Introns were removed from *g6pd* and *white* sequences and the extremities of all protein coding sequences were trimmed to be in codon frame. Alignments for protein coding genes were re-aligned with the Geneious translation alignment program. In addition to gene specific alignments a concatenated dataset was generated in the following order: *COI*-*COII*-*ND5*-*CYTB*-*18S*-*28S*-*g6pd*-*white*. The number of informative sites was calculated using MEGA4^60^.

### Phylogenetic analysis

We used DNA sequences of partial regions of the coding sequence of mitochondrial genes (*COI*, *COII, ND5 and CYTB*) and nuclear ones (*18S*, *28S*, *g6pd* and *white*) from the 19 mosquito species plus *Chagasia bathana*. Phylogenetic analysis of the concatenated, five-partition data set was performed by maximum likelihood and by Bayesian inference. Details of phylogenetic analysis are given in Supplementary Information.

### *Cellia* and *Anopheles* species distribution mapping

Total numbers of mosquito species belonging to the *Cellia* or *Anopheles* subgenera per country were taken from the Walter Reed Biosystematics Unit (WRBU, http://www.wrbu.org/). Data were further represented on maps created with R^61^ using the “sp” package^62^.

### Analysis of the secondary follicle detachment in virgin and mated non-blood fed females

Males and females were separated upon emergence to keep females virgin. A portion of females were put in a cage with males to obtain mated females. After 7 days (time at which all species were at the previtellogenic resting stage, *i.e*. being competent to develop eggs in response to a blood meal), female ovaries were dissected, and the spermatheca of mated females checked for presence of spermatozoa. Ovaries from each female were then mounted on a slide in mounting media and observation of the secondary follicle detachment was performed using a transmitted light microscope. 30 to 60 females were analysed per species and per condition. Data were subjected to Chi-square test. For *An. stephensi*, ovaries from either virgin or mated females were fixed with 4% paraformaldehyde, washed 3 times with PBS-0.05% Tween 20 (PBS-T) and stained with DAPI. After 4 washes with PBS-T, ovaries were mounted on a slide in mounting media. Pictures were taken using a SP5 Leica confocal microscope.

### Analysis of egg development in virgin and mated blood fed females

Virgin and mated females were prepared as described above. A portion of females were put in a cage with males to obtain mated females. On 7 days PE, females were allowed to blood feed on anesthetized mouse or rabbit (Supplementary Table 2). *An. gambiae* and *An. albimanus* females were also fed with fresh human blood (ICAReB Platform, Center for Translational Research, Institut Pasteur, Paris, France) for comparison with mouse-fed mosquitoes. Ovaries were dissected 48 hours after blood feeding, and the total number of eggs in virgin and mated females was counted. Mated status was verified by observing spermatozoa in the spermatheca and only females with a filled spermatheca were taken into account. 30 to 60 females were dissected per species and per condition. Data were subjected to Mann-Whitney non-parametric test.

### Ethical compliance

This study complied with all relevant ethical guidelines and regulations. Project (n° 2013-0132) approved by the Ministère de l’Enseignement Supérieur et de la Recherche – Direction Générale pour la Recherche et l’Innovation – Secrétariat « Autorisation de projet » − 1, rue Descartes, 75231 PARIS cedex 5. Human blood was obtained from ICAReB Platform, Center for Translational Research, Institut Pasteur, Paris, France.

## Supplementary information

Supplementary Information

## Data Availability

The sequence data reported in this paper are tabulated in the Supplementary Information and archived at Genbank.
