# Supplementary material for: Evolution of sexually-transferred steroids and mating-induced phenotypes in Anopheles mosquitoes
Source: Sci Rep. 2019 Mar 15;9:4669. doi: 10.1038/s41598-019-41094-4 (PMC6420574; doi:10.1038/s41598-019-41094-4)
Supplement: Supplementary file 1 — Supplementary Information [file 41598_2019_41094_MOESM1_ESM.pdf]

Evolution of sexually-transferred steroids and mating-induced phenotypes in *Anopheles* mosquitoes

Emilie Pondeville[1,2*], Nicolas Puchot[1,3], Michael Lang[4], Floriane Cherrier[1], Francis Schaffner[5,6], Chantal Dauphin-Villemant[7], Emmanuel Bischoff[1,8], Catherine Bourgouin[1,3*]

Supplementary material and methods

Mosquito species and strains

An. gambiae form M, now called *An. coluzzii* (N'Gousso strain), *An. stephensi* (Sda 500) were permanently reared at Institut Pasteur (France). *An. albimanus* STECLA (MRA-126), *An. arabiensis* DONGOLA (males generously prepared by T. Bukhari and MRA-856), *An. quadrimaculatus* ORLANDO (MRA-139), *An. minimus* MINIMUS1 (MRA-729), *An. dirus* WRAIR2 (MRA-700), *An. farauti* FAR1 (MRA-489), *An. freeborni* F1 (MRA-130), *An. atroparvus* EBRO (MRA-493), *An. quadriannulatus* SANGWE (MRA-1155) and *An. merus* OPHANSI (MRA-803), were obtained through BEI Resources, NIAID, NIH, and contributed by M.Q Benedict (MRA-126,-856,-139,-729,-700,-489,-130,-493), C. Aranda (MRA-493), W. Takken (MRA-1155) and R. Mahraj (MRA-803). *Ae. aegypti* (Liverpool strain) and *Ae. albopictus* (Ho Chi Min Ville, Vietnam) were a generous gift from A-B. Failloux (Institut Pasteur, France). Eggs from *Cx. pipiens* (anautogenous strain) were kindly provided by M. Weill (ISEM, Montpellier, France).

Phylogenetic analysis

The combined dataset had 4398 positions, including 2602 constant positions, 1776 variable positions and 1356 parsimony informative positions. Phylogenetic analysis of the concatenated, five-partition data set was performed by maximum likelihood in PhyML (1) and by Bayesian inference in BEAST (2). For all analyses, partition specific models of nucleotide substitution were selected using the Akaike Information Criterion as calculated in jModelTest 2.1.3 (3, 4). Maximum likelihood inference was done on the concatenated dataset in PhyML using a GTR+I+G model of nucleotide substitution. Node support was determined by performing 100 bootstrap replicates. Bayesian phylogenetic analysis was performed with

BEAST v1.7.5 on the concatenated data set using five partitions with unlinked models of nucleotide substitution (Supplementary Table 5). The partitions corresponded to a single partition for mitochondrial sequences (*COI, COII, ND5* and *CYTB*) and four more partitions for the genes *18S, 28S, g6pd*, and *white*. Mitochondrial genes were combined into one partition because they are closely linked in the mitochondrial genome and largely evolve as a single unit with little to no recombination (5). We used a common strict clock model and a yule birth process as tree prior. While the Bayesian approach places the species of the subgenus *Nyssorhynchus* as sister group of the *Anopheles* subgenus species, they formed the outgroup of the *Anopheles* and *Cellia* lineages in the maximum likelihood approach. Overall, the Bayesian phylogeny revealed high posterior probabilities for each node (>0.9) while the maximum likelihood analysis lacked strong statistical support at the nodes that separate the lineages of the three *Anopheles* subgenera. For species divergence time estimates, we therefore used the Bayesian phylogenetic analysis. We assigned calibration fossil ages to set priors for most recent common ancestors (MRCA). We used *Cx. winchesteri* (33,9-55,8 Ma) (6) (see also http://mosquito-taxonomic-inventory.info/category/fossil-culicidae/fossil-culicidae) to approximate the age of the most recent node shared by *Cx. pipiens* – and *Ae. aegypti*/ *Ae. albopictus*; and *An. dominicanus* (33,9-40,4 Ma) (7, 8) to estimate the age of the most recent node shared by *Anopheles* species. MRCA priors that incorporate fossil calibration dates were assumed to follow an exponential distribution, in the two above mentioned cases with an offset of 33.9 Ma and a mean according to the mean ages of the fossils. Based on a recently published phylogeny of Kamali *et al.* (9), the root age of the *Culicidae* was set to 147 Ma and the prior was assumed to be normally distributed with a standard deviation of 20 Ma. The Markov-Chain Monte-Carlo (MCMC) run was performed with a chain length of 10^8 generations and was recorded every 1000 generations. Estimates were computed with Tracer version 1.5 (http://tree.bio.ed.ac.uk/software/tracer/) and MCMC output analysis was done using TreeAnnotator (2). The first 2000 sampled trees were discarded as the burn-in. The phylogeny was visualized and annotated with Figtree version 1.4 (http://tree.bio.ed.ac.uk/software/figtree/).

Supplementary Figures and Tables

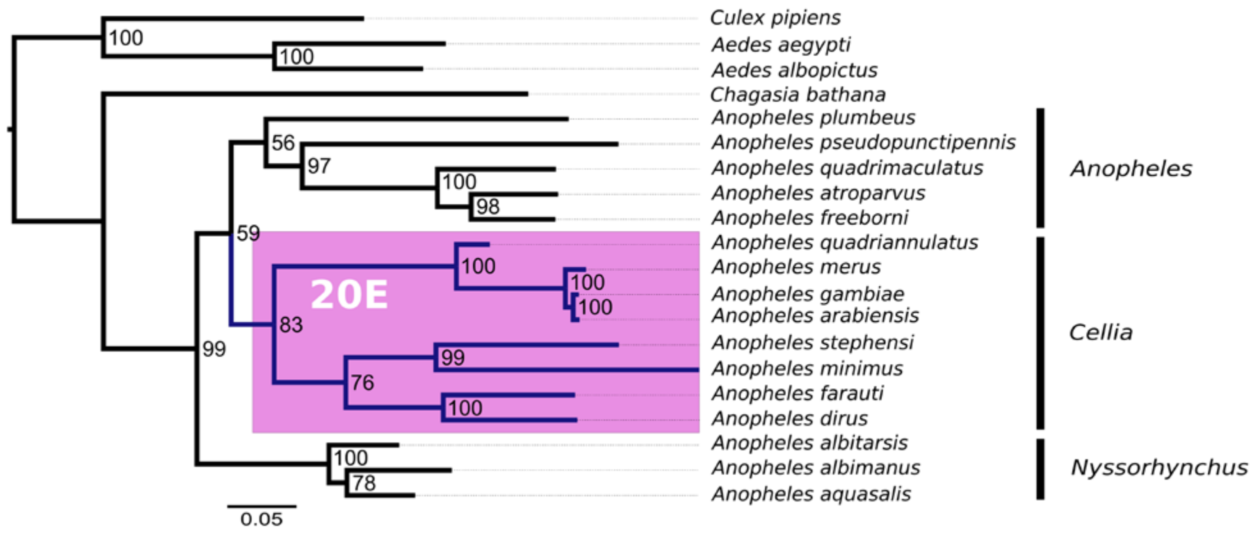

Supplementary Figure 1. Phylogenetic relationships of the genus *Anopheles* and evolution of male 20E production. Maximum likelihood phylogeny (PhyML) based on a concatenated dataset. Bootstrap supports (100 replicates) are presented on the right side of each node. Bars on the right side indicate species that belong to the same subgenus. The lineages of the subgenus *Cellia* are highlighted in blue. The lineages with male 20E production are shaded in pink.

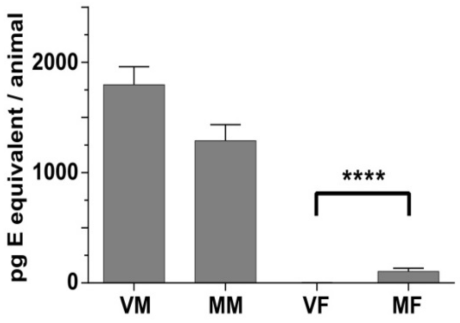

Supplementary Figure 2. *Anopheles stephensi* males transfer ecdysteroids to females during mating. Ecdysteroid titers were measured in virgin males (VM), in mated males (MM) just after copulation, in virgin females (VF), and in mated females (MF) just after copulation. Ecdysteroids were extracted from each individual mosquito and quantified by EIA. Results are expressed as mean +/- SEM in in pg E equivalents per animal. Results were subjected to statistical analysis using Mann-Whitney test (****, P< 0.0001).

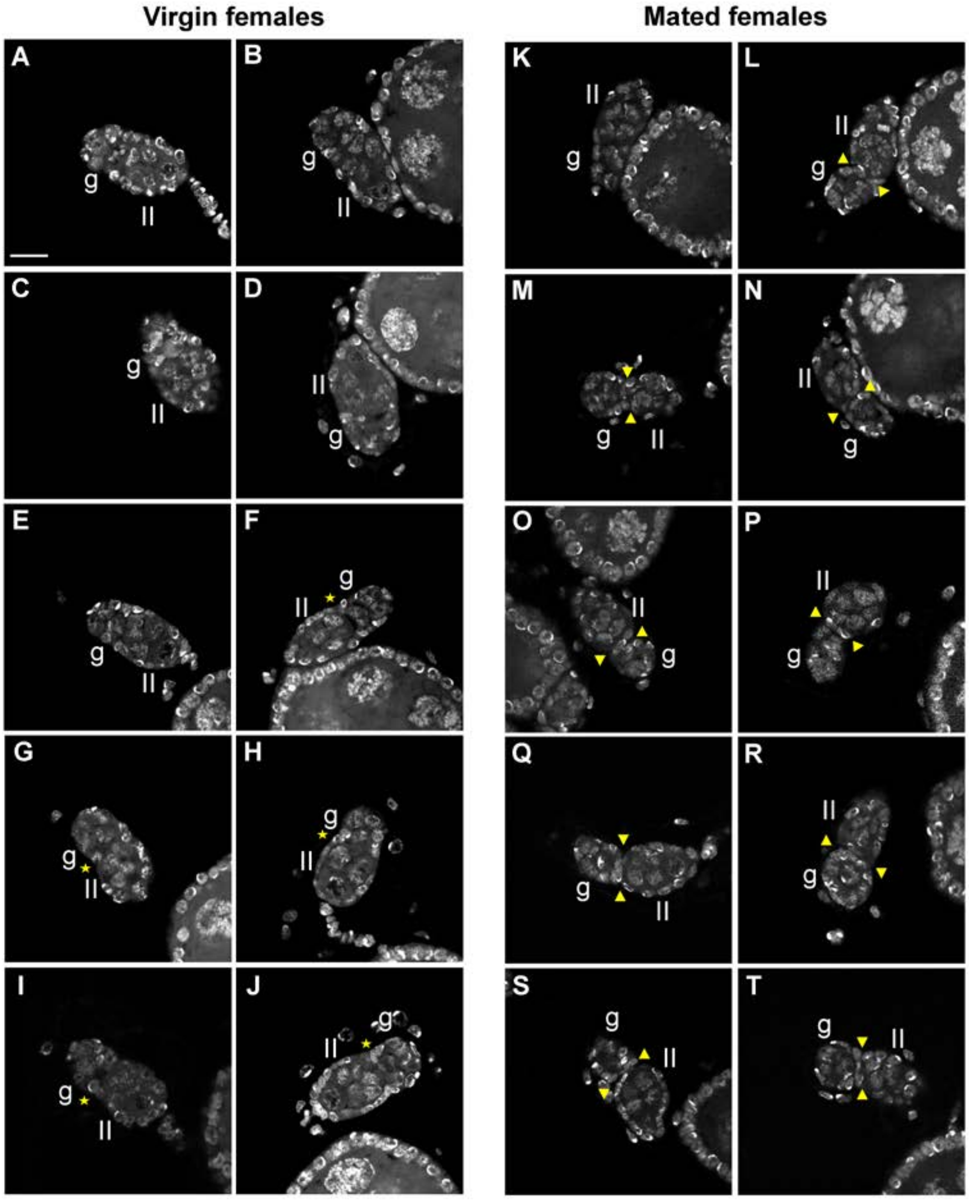

Supplementary Figure 3. Secondary follicle detachment from the germarium in *Anopheles stephensi* non blood-fed females according to the insemination status. Confocal pictures of ovarioles showing the germarium and the secondary follicle from 10 non blood-fed females either virgin (A to J) or mated (K to T) stained with DAPI. g: germarium, II: secondary follicle. Yellow arrowheads show secondary follicles detached from the germarium and yellow stars show secondary follicles in progress of detachment. Follicles not marked are not detached. Scale bar is 10.25 µm.

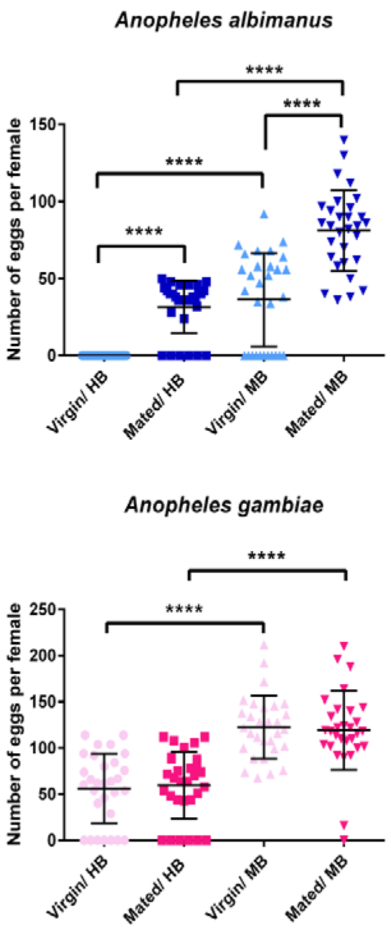

Supplementary Figure 4. Egg development in *Anopheles albimanus* (*Nyssorhynchus* subgenus) and *Anopheles gambiae* (*Cellia* subgenus) virgin and mated females fed on mouse or human blood. Total number of eggs in virgin (light colours) and mated (dark colours) females was counted 48 hours after feeding on either human blood (HB) or mouse blood (MB). *An. albimanus* is coloured in blue and *An. gambiae* in pink. Data were subjected to Mann-Whitney non-parametric test. Mated females from *An. albimanus* develop significantly more eggs then virgin females when fed on human blood (Mann-Whitney U=90, p<0.0001) or mouse blood (Mann-Whitney U=122, p<0.0001). Virgin and mated females of both species develop significantly more eggs when fed on mouse blood compared to human blood (*An. albimanus* virgin: Mann-Whitney U=165, p<0.0001; *An. albimanus* mated: Mann-Whitney U=52.50, p<0.0001; *An. gambiae* virgin: Mann-Whitney U=74, p<0.0001; *An. gambiae* mated: Mann-Whitney U=90, p<0.0001).

Node	Taxon Set	Divergence Time*	Node	Taxon Set	Divergence Time*
1	all	124.2 Ma (83.7-165.8)	11	*An. atroparvus – An. quadrimaculatus*	25.6 Ma (16.7-34.8)
2	*Aedes – Culex* (genus)	78.9 Ma (51.6-106.5)	12	*An. atroparvus – An. freeborni*	18.1 Ma (11.5-24.7)
3	*Aedes* (genus)	35.6 Ma (23.1-48.3)	13	*Cellia* (subgenus)	69.2 Ma (45.6-93.0)
4	*Anopheles – Chagasia* (genus)	113.7 Ma (75.2-152.4)	14	*An. farauti – An. stephensi*	62.5 Ma (41.1-84.3)
5	*Anopheles* (genus)	84.1 Ma (55.8-112.7)	15	*An. stephensi – An. minimus*	46.6 Ma (30.0-62.8)
6	*Nyssorhynchus – Anopheles* (subgenus)	76.4 Ma (50.4-102.3)	16	*An. farauti – An. dirus*	31.6 Ma (20.5-42.9)
7	*Nyssorhynchus* (subgenus)	20.7 Ma (13.5-28.3)	17	*An. gambiae – An. quadriannulatus*	17.2 Ma (11.0-23.7)
8	*An. aquasalis – An. albitarsis*	17.4 Ma (11.1-24.1)	18	*An. gambiae – An. merus*	3.2 Ma (1.9-4.5)
9	*Anopheles* (subgenus)	71.9 Ma (47.7-97.0)	19	*An. gambiae – An. arabiensis*	1.2 Ma (0.6-1.9)
10	*An. atroparvus - An. pseudopunctipennis*	61.9 Ma (40.3-82.9)			

*Estimates are the posterior means with 95% highest posterior density intervals.

Supplementary Table 1. Divergence time estimates of the *Anopheles* species and outgroups. Estimates were obtained from the Bayesian phylogenetic analysis and are based on fossil data for temporal calibration.

***Anopheles* species**	**Animal species**	**Blood source**
An. quadrimaculatus	mouse	
An. atroparvus	rabbit	
An. freeborni	mouse	
An. albimanus	mouse	human
An. stephensi	mouse	
An. minimus	mouse	
An. dirus	rabbit	
An. farauti	mouse	
An. merus	mouse	
An. gambiae	mouse	human
An. arabiensis	rabbit	
An. quadriannulatus	mouse	

Supplementary Table 2. Animal species or blood source on which mosquitoes were fed in this study.

Name	Sequence	Locus	Reference
COI-2	TCCATTGCACTAATCTGCCA	*COI*	(10)
LepF1	ATTCAACCAATCATAAAGATATTGG		(11)
tLEU-2	ATGGCAGATTAGTGCAATGA	*COII*	modified from (12)
tLys-2	TGATTTAAGAGATCATTACTTG		modified from (12)
T7-ND5	TAATACGACTCACTATAGGGATTAACTGTATGTTATTCITTYC	*ND5*	modified from (13)
tRNAPhe-2	CCTAACATCTTCAGTGTCATGCT		modified from (13)
cytb-R2	TACTGGTCGAGCTCCAATTCA	*CYTB*	this study
T7-cytbF	TAATACGACTCACTATAGGGACAAATATCATTTTGAGGAGCIACAG		modified from (14)
18S-F2	CAGCTCCACTAGCGTATATTAA	*18S*	this study
18S-R2	TTAACCAGACAAATCIATCCAC		this study
D2F2	AGTCGTGTTGCTTGATAGTG	*28S*	(15)
D2R2	CTTGGTCCGTGTTTCAAGAC		(15)
T7-G6pdF1	TAATACGACTCACTATAGGGTGGACACGGARGGNACICAYTTYGA	*g6pd*	this study
T7-G6pdF2	TAATACGACTCACTATAGGGTCGGGAGATTTGGCTAAIAARAARATHTA		this study
G6pdR1	TGTTCCAGGTAGGGCTRAANATYTGRTT		this study
G6pdR2	CATCAGGTTYTGNACCATYTC		this study
T7-wF1	TAATACGACTCACTATAGGGCGGCTCMGIAAYTGYTGYAC	*white*	this study
T7-wz2E-2	TAATACGACTCACTATAGGGTACAACCCGGCNGAYTTYTA		modified from (16)
wR1	CCTCGACGCGGAARTTRAANGTYTC		this study
wR2	GGAACCAGGACAGGTACGANAIRTAYTTRAA		this study

Supplementary Table 3. Sequences of primers used to sequence DNA for the phylogenetic analyses. Partial genomic regions of four nuclear genes (*g6pd*, *white*, *18S* and *28S*) and four mitochondrial genes (*COI*, *COII*, *ND5* and *CYTB*) were amplified by PCR with gene-specific or degenerate primers. Degenerate oligonucleotides were designed based on previous studies and optionally modified or newly designed. Some oligonucleotide sequences contained T7 or SP6 universal primer sequences at their 5' end, following Bonacum *et al.* (17).

Species	Locus COI	COII	ND5	CYTB	18S	28S	g6pd	white
Aedes aegypti	NC010241	NC010241	NC010241	NC010241	AAEL017915	AAEL017581	XM001660122	U73826
Aees albopictus	AY072044	AY072044	AY072044	AY072044	X57172	MQSRNAGN	---	U73828
Anopheles albimanus	AF417695	AF417731	AF311270	AF311251	MSQINSP	MSQINSP	AF317824	MSQWHITE
Anopheles albitarsis	HQ335344	HQ335344	HQ335344	HQ335344	AF417768	AF417803	AF317823	AF318198
Anopheles aquasalis	AF417697	**MG560168**	**MG560169**	**MG560174**	AF417769	AF417804	**MG560181**	***MG560185***
Anopheles arabiensis	AF417705	AF417741	SRS008420 contigs: 7466, 23167, 23406, 36079, 47611	SRS008420 contigs: 1529, 8012, 23026, 33951, 34964, 44097	AF417777	AF417812	AY118019	AARA010493-RA
Anopheles atroparvus	**MG560162**	**MG560165**	**MG560170**	**MG560175**	AM072973	**MG560178**	**MG560184**	***MG560187***
Anopheles dirus	JX219731	JX219731	JX219731	JX219731	AF417779	AF417814	ADIR005618-RA	ADIR001487-RA
Anopheles farauti	AF417708	AF417744	JX219741	HQ840893	AF121054	AF417815	AFAF008422-RA	AXCN01000101.1 pos. 115279-116093
Anopheles freeborni	AF417717	AF417753	SRS008481 contigs: 12850, 20811, 21808, 23967	SR008481 contigs: 18, 9086, 23902, 24406, 26852, 29845, 41851	AF417788	AF417824	---	AFU73830
Anopheles gambiae	MSQMTCG	MSQMTCG	MSQMTCG	MSQMTCG	AM157179	KC177663	AGAP012678	AGAP000553
Anopheles merus	**MG560163**	**MG560167**	**MG560171**	**MG560176**	**MG560177**	**MG560180**	**MG560182**	***MG560186***
Anopheles minimus	AF417710	AF417746	---	KF431913	AF417781	DQ523567	AMIN009223-RA	AMIN005306-RA
Anopheles plumbeus	**MG560164**	**MG560166**	**MG560172**	**MG560173**	AM072974	**MG560179**	**MG560183**	**---**
Anopheles pseudopunctipennis	AF417721	AF417757	AF311272	---	AF417792	AF417828	AF317810	AF318197
Anopheles quadriannulatus	DQ792581	DQ792581	SRS008482 contigs: 9810, 9922, 12564	SRS008482 contigs: 2436, 3560, 5140, 6641, 12944, 25648, 35018	---	KB667953 genomic scaffold supercont1.758 pos 16103-16634	AY118023	AQUA010818-RA
Anopheles quadrimaculatus	NC000875	NC000875	NC000875	NC000875	AY988423	AY569555	AF317809	AF318207
Anopheles stephensi	AF417713	AF417749	AF311273	AF311254	AF417784	AF417820	AF317808	AF318208
Chagasia bathana	AF417726	AF417762	AF311281	AF311253	AF417797	AF417831	AF317819	AF318194
Culex pipiens	NC015079	NC015079	NC015079	NC015079	AY988445	---	CPU09034	---

Footnote: Genbank Accession Numbers, VectorBase identifiers (shaded grey), or BlastN search hits at VectorBase (shaded dark grey). Newly generated sequences are highlighted in bold.

Supplementary Table 4. GenBank Accession Numbers of the dataset used for the phylogenetic analyses.

Partition, site model, gamma categories, nucleotide frequencies

COI-COII-ND5-CYTB	*18S*	*28S*	*g6pd*	*white*
GTR+I+G	TN93+I+G	TN93+I+G	TN93+I+G	GTR+I+G
5	5	5	5	5
estimated	estimated	estimated	estimated	estimated

Supplementary Table 5. Partition specific site models and parameters.

References

1. Guindon S, Gascuel O. A simple, fast, and accurate algorithm to estimate large phylogenies by maximum likelihood. Syst Biol. 2003;52(5):696-704.
2. Drummond AJ, Suchard MA, Xie D, Rambaut A. Bayesian phylogenetics with BEAUti and the BEAST 1.7. Mol Biol Evol. 2012;29(8):1969-73.
3. Darriba D, Taboada GL, Doallo R, Posada D. jModelTest 2: more models, new heuristics and parallel computing. Nat Methods. 2012;9(8):772.
4. Posada D. jModelTest: phylogenetic model averaging. Mol Biol Evol. 2008;25(7):1253-6.
5. Ballard JW. Comparative genomics of mitochondrial DNA in members of the *Drosophila melanogaster* subgroup. J Mol Evol. 2000;51(1):48-63.
6. Cockerell TDA. The oldest mosquitoes. Nature. 1919;103:44.
7. Zavortink TJ, Poinar JR GO. *Anopheles* (*Nyssorhynchus*) *dominicanus* sp. n. (Diptera: Culicidae) from Dominican Amber. Ann Entomol Soc Am. 2000;93(6):1230–5.
8. Borkent A, Grimaldi DA. The Earliest Fossil Mosquito (Diptera: Culicidae), in Mid-Cretaceous Burmese Amber. Ann Entomol Soc Am. 2004;97(5):882–8.
9. Kamali M, Marek PE, Peery A, Antonio-Nkondjio C, Ndo C, Tu Z, et al. Multigene phylogenetics reveals temporal diversification of major African malaria vectors. PloS one. 2014;9(4):e93580.
10. Wang BC, Park J, Watabe HA, Gao JJ, Xiangyu JG, Aotsuka T, et al. Molecular phylogeny of the *Drosophila virilis* section (Diptera: Drosophilidae) based on mitochondrial and nuclear sequences. Mol Phylogenet Evol. 2006;40(2):484-500.
11. Hajibabaei M, Janzen DH, Burns JM, Hallwachs W, Hebert PD. DNA barcodes distinguish species of tropical Lepidoptera. Proc Natl Acad Sci USA. 2006;103(4):968-71.
12. Liu H, Beckenbach AT. Evolution of the mitochondrial cytochrome oxidase II gene among 10 orders of insects. Mol Phylogenet Evol. 1992;1(1):41-52.
13. Krzywinski J, Wilkerson RC, Besansky NJ. Evolution of mitochondrial and ribosomal gene sequences in Anophelinae (Diptera: Culicidae): implications for phylogeny reconstruction. Mol Phylogenet Evol. 2001;18(3):479-87.
14. Lyman DF, Monteiro FA, Escalante AA, Cordon-Rosales C, Wesson DM, Dujardin JP, et al. Mitochondrial DNA sequence variation among triatomine vectors of Chagas' disease. Am J Trop Med Hyg. 1999;60(3):377-86.

15. Sallum MAM, Schultz TR, Foster PG, Aronstein K, Wirtz RA, Wilkerson RC. Phylogeny of Anophelinae (Diptera: Culicidae) based on nuclear ribosomal and mitochondrial DNA sequences. Syst Entomol. 2002;27:361-82.
16. Zwiebel LJ, Saccone G, Zacharopoulou A, Besansky NJ, Favia G, Collins FH, et al. The *white* gene of *Ceratitis capitata*: a phenotypic marker for germline transformation. Science. 1995;270(5244):2005-8.
17. Bonacum J, DeSalle R, O'Grady P, Olivera D, Wintermute J, Zilversmit M. New nuclear and mitochondrial primers for systematics and comparative genomics in Drosophilidae. Dros Inf Serv. 2001;84:201-4.
